# 536 Objective Quantification of Hypertrophic Scar and Donor Scar between 2 to 7 months Post-burn Injury

**DOI:** 10.1093/jbcr/irac012.165

**Published:** 2022-03-23

**Authors:** Zoë Edger-Lacoursière, Elisabeth Marois-Pagé, Ana de Oliveira, M Réadaptation, Valérie Calva, Bernadette Nedelec

**Affiliations:** Centre professionnel d'Ergothérapie and McGill University, Montréal, Quebec; Hôpital de Réadaptation Villa Medica, Outremont, Quebec; CRCHUM, Montreal, Quebec; Hôpital de réadaptation Villa Medica, Montréal, Quebec; Villa Medica Rehabilitation Hospital, Longueuil, Quebec; McGill University, Montreal, Quebec; McGill University, Montreal, Quebec

## Abstract

**Introduction:**

Very few objective scar evaluations have been conducted with the burn survivor population, which limits our knowledge of the clinical recovery profile of hypertrophic scars (HSc) and donor site scars (D), thus having an impact on rehabilitation intervention and treatment prioritization. The purpose of this study was to prospectively quantify the thickness, pliability, erythema and pigmentation of post-burn HSc, donor sites (normal scar) and normal skin in different anatomical locations between 2 and 7 months post-burn using objective instrumentation. A secondary objective was to compare this data with anatomic-specific normative data using the same objective instrumentation.

**Methods:**

Skin characteristics of HSc, D and N in 44 burn survivors were measured at 2, 3, 4, 5, 6 and 7 months post-burn using validated instrumentation: high-frequency ultrasound for thickness, Cutometer® to measure pliability and Mexameter® to measure erythema and pigmentation. Up to five sites were assessed on the same participant if their scar was located on the upper extremity (UE), lower extremity (LE) and trunk. A mixed model two-way analysis of variance was used to investigate the differences in means between sites at each time point and between time points at each site.

**Results:**

The results revealed that HSc sites were thicker than D and N at all time points and UE and trunk HSc were thicker than LE HSc at 7 months post-burn, pliability of trunk HSc did not improve over time, and UE HSc was more erythematous at 7 months compared to other anatomical sites whereas D erythema decreases from 2 to 7 months.

**Conclusions:**

Scar management treatments should prioritize the UE and trunk sites which developed HSc during the first two months post-burn and continues to vary significantly from normal scar and normal skin at 7 months. Furthermore, these results provide preliminary evidence that the recovery profile of HSc varies at different anatomical sites and that thickness is the characteristic that distinguishes HSc from normal scar and normal skin.

